# 10 Practical Considerations for the Conduct of Multi‐National/Database Studies in Pharmacoepidemiology

**DOI:** 10.1002/pds.70203

**Published:** 2025-08-31

**Authors:** Kenneth K. C. Man, Anton Pottegård

**Affiliations:** ^1^ Research Department of Practice and Policy, School of Pharmacy University College London London UK; ^2^ Clinical Pharmacology, Pharmacy and Environmental Medicine, Department of Public Health University of Southern Denmark Odense Denmark

**Keywords:** common analytics, data harmonization, multi‐database, multi‐national/database, real‐world data

## Abstract

**Background:**

Multi‐national/database pharmacoepidemiological studies are increasingly used to address questions that require pooled evidence across populations but introduce challenges in design, harmonization, and analysis.

**Objective:**

To share 10 practical considerations and common pitfalls in planning, executing, and reporting multi‐national/database studies, with strategies to mitigate them.

**Approach:**

Practical guidance article synthesizing experience from multi‐national/database projects and literature‐based exemplars; no original data collection.

**Results:**

We summarize ten considerations spanning: protocol development; follow‐up time and eligibility assessment; data harmonization (including thorough metadata and master mapping tables); feasibility checks within each source; statistical model diagnostics; and transparent reporting. We illustrate how local clinical practices, coding systems, and reimbursement policies can shape outcomes and interpretation, and we emphasize the need for proactive, consistent communication among collaborators to ensure aligned implementation.

**Conclusion:**

Multi‐national/database studies are complex but feasible with structured planning, clear communication, and proactive problem‐solving. Adopting the outlined practices can reduce avoidable heterogeneity and improve the robustness and interpretability of findings.

**Summary**

- Multi‐national/database pharmacoepidemiology studies require careful planning and coordination due to differences in data sources, coding systems, and healthcare practices across regions.
- We highlight ten critical focus areas, such as metadata tables, master mapping tables, and crude rates, which are essential for ensuring consistency in data harmonization and interpretation across sites.
- Detailed follow‐up time reporting, including both on‐treatment and total follow‐up, is vital for understanding patient retention and treatment discontinuation.
- A clear and fully agreed‐upon protocol, combined with regular communication, ensures consistent study implementation and interpretation across data sources.
- Recommendations are provided for conducting model diagnostics, including propensity score balance checks and failure plots, to ensure robust and comparable results across participating sites.

## 1 Introduction

Multi‐national/database studies with distributed network approaches are increasingly common in pharmacoepidemiology. Such studies allow researchers to address questions where the involvement of multiple data sources is necessary to attain sufficient statistical precision due to a rare exposure or outcome, questions related to variation in effect sizes for example, across ethnicities, or questions where documentation of variation in treatment patterns across countries or regions is the focus [1].

Performing pharmacoepidemiological studies is always complex, not only regarding the study design and planning, but also in study implementation. This complexity is markedly increased when doing multi‐national/database studies compared to studies based on a single data source, from time to data access and ethics approval, data diversity across sources, and standardizing evidence [1].

With this paper, we, as proponents of multi‐national collaboration in pharmacoepidemiology, aim to provide suggestions on avoiding common pitfalls in the practical implementation of a pharmacoepidemiological outcome study across multiple countries. The areas described have been selected based on our practical experience with the conduct of such studies and all constitute areas within which we have made mistakes that cost us both time, effort, and frustration. This extends a recent Core Concept paper in pharmacoepidemiology regarding multi‐database studies [1], which describes the technical and conceptual aspects. We recommend reading that paper as an introduction to the concepts of multi‐database studies, as we focus more on practical issues.

The focus areas are all relevant regardless of whether a study is implemented on a ‘common protocol’ approach, where each site develops its own analytical code, or the ‘common program/analytics’ approach, in which analytical code developed by a coordinating entity is distributed to collaborators. While the use of common data models (CDM), such as OMOP, and associated analytic platforms is an important strategy for harmonizing diverse databases, our intention here is not to prescribe any single CDM or platform. Instead, we offer a general overview of different approaches that have been used in practice. Some focus areas are, however, particularly important for studies following a ‘common protocol’ approach, as they allow the principal investigator to assess aspects of study implementation that might have been incorrectly carried out by the individual programmers. Further, while the paper is focused on multinational/database outcome studies, some of the focus areas might also prove helpful for the junior researcher doing an outcome study in a single database or for purely descriptive studies when involving multiple countries.

We describe 10 focus areas, which we have divided into three parts, corresponding to protocol development, analysis, and reporting.

## 2 Protocol Development

### 2.1 Meta Data Table

A meta data table provides information about the datasets being used, such as data source nature, population characteristics, data collection methods, coding systems (e.g., ICD codes, ATC codes), and data quality indicators.

As a coordinator, it is advisable to ask your data partners to provide a detailed meta data table that gives you a clear picture of what data the group is working on. There are standard templates, for example, the one recently proposed by Pajouheshnia et al. [2], which may be helpful. Regardless of the template, a meta data table should include the time (time periods covered, lag time), population characteristics (rules for inclusion and exclusion criteria), types of available data (e.g., prescriptions, diagnoses, lab results), sources of data (e.g., sectors or contact types covered) and any known limitations or biases of the data (including quality and validations on the content). A previous scoping review similarly recommended reporting these elements and identified nine key metadata dimensions to ensure reproducibility [3]. The generic information should ideally be annotated with issues of particular relevance for the specific study question, for example, results of validation efforts for parameters to be used. It would also be informative if each collaborating site could include the healthcare system and culture related to the database, which may have an impact on the data generation process or shed light on any inconsistency that may occur at the later stage of the study. Understanding these aspects is essential for assessing the feasibility of pooling data and interpreting the results.

Providing a thorough database description has several benefits not only for the planning, conduct, and interpretation of the current study but also for future research, as a well‐structured database description can be reused in future projects. For instance, the HARPER protocol [4] template requires detailed information about the databases when drafting a new study protocol. Having a comprehensive, ready‐made database description makes it significantly easier to incorporate “new” databases into ongoing or future studies.

*Example*:

In a study investigating the association between oral fluoroquinolones and retinal detachment using data from Hong Kong and Taiwan [5], we initially overlooked key differences between the two databases. The Hong Kong database was an electronic health record (EHR) system, while the Taiwanese database was claims‐based. This difference impacted the coding of our event of interest. In the EHR system, a diagnosis is typically recorded once per episode and not repeatedly coded for follow‐up visits. In contrast, in the claims database, the same diagnosis code can appear multiple times, even during follow‐up visits. This led to an inconsistency in how retinal detachment cases were captured. As a result, we had to revise our event definition, shifting from diagnosis codes to procedure codes to ensure more accurate outcome ascertainment across both databases.

### 2.2 Master Mapping Table

A master mapping table describing codes used to define exposures, outcomes, and covariates is necessary to ensure consistent implementation of a study across datasets. Drug coding (e.g., ATC codes vs. BNF codes) and diagnostic coding (e.g., different ICD versions) often vary by data source. As a coordinator, you should ensure that such a table is created and versioned, with each update documented and agreed upon by all collaborators.

When developing the master mapping table, it may be helpful to differentiate between mapping codes and mapping concepts. Mapping codes, such as harmonizing ICD‐10 codes to SNOMED, can be more straightforward if a standardized crosswalk between coding systems is available. However, this is only sometimes the case, and challenges arise when participating data sources use different and less compatible coding systems. For example, databases like CPRD in the UK use the Read and SNOMED codes, while databases in Nordic countries typically rely on ICD codes [6]. These differences complicate the mapping process, as direct code‐to‐code conversions may not always capture the nuances of clinical concepts. In such cases, condition‐specific algorithms can be invaluable. For key parameters, such as definitions of the outcome events, it will often be relevant to further consult local clinicians to understand coding practices, in particular in situations where the outcome diagnoses have not been formally validated. In addition to supporting implementation, presenting the master mapping table with the research publication is critical to transparently convey how conditions were defined and harmonized and also supports future research by providing such mappings.

Striking a balance between protocol harmonization and study quality is particularly challenging in multi‐national/database research. For instance, one site might only have hospital records, while another might have relatively complete medical records. For some variables, the decision to harmonize protocols by limiting data usage or optimizing methods for each database may affect study validity. In such cases, using the best available methods for each site may be preferable while transparently documenting differences in approach. This ensures that diversity in the study design reflects real‐world differences in healthcare systems rather than unnecessary compromises.

*Example*:

In a study examining gastrointestinal bleeding (GIB) risks with rivaroxaban versus aspirin in patients with atrial fibrillation [7], we used ICD codes in Hong Kong data to define atrial fibrillation (AF). However, when it came to implementing the same definition in UK data, the process was delayed as it became much more labor‐intensive due to the need to identify equivalent Read codes for the conditions. A well‐constructed mapping table, or even better a machine‐readable format, could significantly simplify this process by documenting crosswalks between different coding systems, ensuring consistency across multiple data sources and reducing the potential for coding errors.

### 2.3 Data Checks

Conducting initial data checks would be a helpful first step in multi‐national/database studies to identify any significant differences that may affect the analysis. These checks include examining the counts of individuals meeting inclusion criteria, follow‐up times, and the completeness of the derived study variables in the master mapping table.

Detailed information on crude counts of individuals eligible for the study and assessing their follow‐up times (mean, median, and distribution) is critical to understanding patient retention and turnover in each database. For example, databases with shorter follow‐up periods may be less suitable for studying long‐term outcomes. Crude counts and follow‐up distributions can reveal feasibility issues early in the study design phase, preventing wasted effort on protocols that cannot be implemented across all sites. Differences in the data sources are expected, and these differences would rather be opportunities to explore real‐world variability. Collaborating with local investigators to understand region‐specific guidelines and practices, such as diagnostic and treatment criteria, provides valuable context for interpreting the results.

Of note, performing such initial data checks including obtaining crude counts is perfectly appropriate even before finalizing the protocol, as also argued by others [8], as they help assess the feasibility of conducting a meaningful analysis and ensure the robustness of the study from the outset.

*Example*:

In a study implementing an active comparator new user cohort design comparing use of GLP‐1 receptor agonists (GLP‐1RA) and DPP‐4 inhibitors (DPP4i) [9], it became apparent that, at one site, reimbursement policies required patients to try DPP4i before being allowed to initiate GLP‐1RA. This effectively prevented the study from maintaining the intended study design, as only very few people entered the GLP‐1RA cohort without prior DPP4i use. Initial counts of individuals meeting the main cohort inclusion criteria would have revealed that it was impossible to implement the study as intended.

### 2.4 Exposure Patterns

After data checks are completed, the next step is to examine in more detail the exposure patterns for key variables, including the number of exposed individuals, treatment switching, and treatment durations.

The leading site may request counts of exposed individuals for each key variable across sites, stratified by time periods or other relevant factors (e.g., age groups or comorbidities), which could help ensure that the study population is well‐represented and that exposure patterns align with the research question. Further, understanding exposure patterns, including characteristics of treatment initiation, switching, and discontinuation rates, could help interpret study results. For example, on‐treatment follow‐up should be reported separately from the total follow‐up to capture discontinuation rates and treatment persistence. This provides insights into variations in utilization behavior and healthcare practices across regions.

*Example*:

In a study that looked into the comparative effectiveness of direct oral anticoagulants (DOAC) [10], we reported both on‐treatment follow‐up and total follow‐up times. By providing these follow‐up times separately for each DOAC cohort and database, we were able to capture critical variations in treatment persistence. In particular, the median on‐treatment follow‐up varied significantly between DOACs and databases, ranging from 4 days for dabigatran in one database to almost 20 months for apixaban in another, suggesting very different patient populations with different treatment discontinuation rates.

### 2.5 Fully Agreed Protocol With Data Harmonisation Plan

A commonly agreed protocol ensures that all collaborators adhere to the same study design, particularly in complex pharmacoepidemiology studies where data harmonization is essential for pooling data across different sources. A good protocol not only serves technical purposes but also ensures a mutual understanding of the overall study conduct, terms covered, and regional differences across participating sites.

Ensure that the protocol includes a comprehensive study design, data harmonization plan, statistical analysis plan, and consider registering it in a public domain, such as the European HMA‐EMA Catalogues of real‐world data sources and studies register or the Open Science Framework Real‐World evidence registry. The protocol can follow recommended templates such as the HARPER protocol template [4] or at least derive inspiration from such templates to ensure all critical components are described sufficiently.

*Example*:

In a study on opioid use, we initially listed the opioid drugs to be included without specifying whether their use should be restricted to analgesic purposes [11]. The term “opioid” was interpreted differently across sites. Consequently, some sites included specific opioids that are used for non‐analgesic purposes, such as treating opioid dependence, while other sites only included opioids used exclusively for pain relief. This could have led to substantial differences had we not recognized and separated these different uses.

## 3 Analysis

### 3.1 Flowchart of Included Participants

Flowcharts are essential for illustrating the process of participant inclusion and exclusion, particularly in multi‐national/database studies where differences in cohort characteristics can mean very different patient selection across sites.

Ensure that each data partner provides a flowchart following a predefined sequence, documenting the number of participants excluded at each stage of the selection process. This should include reasons for exclusion, such as lack of follow‐up data or failure to meet specific inclusion criteria.

*Example*:

In a multi‐national/database study [12], each site implemented the exclusion in different orders. When a reviewer later requested a combined flowchart of participant inclusion and exclusion, it was impossible to generate this without redoing the cohort creation process at multiple sites, underscoring the importance of establishing a predefined, uniform sequence for applying inclusion and exclusion criteria across all sites.

### 3.2 Description of the Study Cohort (Table 1)

Descriptive data on the study cohorts (commonly reported as Table 1) provide an overview of the study cohort's characteristics. This allows for comparison across databases and helps in identifying any potential sources of bias or other variations. For instance, differences in age distribution or comorbidity prevalence could affect the generalizability of the findings.

When creating the table, present both counts and proportions for each variable, split into two columns. This allows for easier comparison across sites and facilitates the later combination of data when conducting pooled analyses.

*Example*:

In a study comparing GIB risks between rivaroxaban and aspirin [7], while the age distribution and sex ratio were similar across the two databases, there were notable differences in the baseline bleeding risk as measured by the HAS‐BLED score. This notable difference in patients' characteristics could potentially explain the differences in the results across sites. This discrepancy underscores the importance of descriptive analyses to contextualize study findings and ensure accurate interpretation of pooled results.

### 3.3 Model Diagnostic

Proper review of diagnostics for statistical models, such as regression output and propensity score (PS) diagnostics, is vital in pharmacoepidemiology [13].

Request diagnostics from your data partners for any models applied. This not only includes assessing the balance of covariates before and after PS matching/weighting, but also regression output from PS estimation, plots of propensity score distributions, and regression output from for example, Cox regressions or logistic regressions. Understanding how well the analysis model has performed in each database is essential for ensuring that the common analytics work properly and that confounding is adequately controlled.

*Example*:

In a multi‐national/database study on adverse effects of glucose‐lowering medication [9], one site reported a marked effect of confounder adjustment, with the propensity score‐based weighing leading to strongly increased risk estimates. A similar effect was not observed at the remaining sites. Upon inspection, it was revealed that some individuals received very high weights, which in turn was caused by clearly separated propensity score distributions. This highlights that fitting a PS model can be an iterative process, especially in a multi‐national/database study where each participating site has different population characteristics and data structures.

### 3.4 Failure Plots (e.g., Kaplan–Meier or Cumulative Incidence Curves)

Failure plots, such as Kaplan–Meier curves, are standard tools in pharmacoepidemiology for visualizing time‐to‐event data.

Ask for these plots from each database to visually inspect the survival distributions and cumulative incidences of the events of interest. These plots help in identifying any deviations or unexpected patterns that could indicate data quality issues or the need for further investigation. Consistency in the shape and pattern of these plots across databases adds credibility to the pooled analysis.

*Example*:

The survival curves provided by some sites indicated that no cases of ADHD occurred within the first 6 years of follow‐up [12]. Upon investigation, it became clear that this was the result of miscommunication regarding the outcome definition. We required at least 6 years of follow‐up time; however, events that occurred within the first 6 years should also be counted.

## 4 Reporting

### 4.1 Quality Control Plan on Transcription From Aggregated Results to Manuscript/Reporting Document and Consensus on the Interpretation of the Results

Implement a thorough quality control plan to ensure that results are accurately transcribed from aggregated data into the final manuscript. This should include checks for transcription errors, consistent reporting of statistics (e.g., outcome metrics, confidence intervals), and overall accuracy in data presentation. To facilitate consistent incorporation of results into tables and figures, the study lead may consider providing partners with a standardized format for reporting, so that the programs to compile and visualize data can be developed and run uniformly across sites.

Additionally, it is critical to confirm that all sites inspect and confirm the transfer of their data and also agree on the interpretation of the results. Misalignment in how data is interpreted or reported across sites can lead to discrepancies in conclusions. To address this, leading sites may establish templates to collect partners' comments throughout the review cycles so that collaborators can systematically provide feedback and resolve any discrepancies. Regular discussions among collaborators to review and agree on the interpretation of key findings will help ensure consistency and coherence in the final report. This step safeguards the study's scientific integrity and fosters a shared understanding of the results among all contributing partners.

Through its publication cycle, multi‐national/database studies can also further enhance their transparency by sharing analytical code [14] and adhering to the FAIR (Findable, Accessible, Interoperable, Reproducible) principles [15], and enabling colleagues from different fields to learn from previous work.

*Example*:

In one of the multi‐national drug utilization studies [16], a series of ad‐hoc quality checks revealed several transcription errors when incorporating results sent from participating sites into the final paper. The ad hoc nature of these checks led to delays in the study submission.

## 5 Discussion

Multi‐national/database pharmacoepidemiological studies offer invaluable insights into drug safety and effectiveness across diverse populations, but they also present unique challenges in study implementation and coordination. In this paper, we have identified and described several areas where the complexity of handling multiple databases, diverse healthcare settings, and different coding systems creates pitfalls if not appropriately addressed. Although this manuscript does not focus exclusively on CDMs, many of the components we discuss, such as detailed code‐mapping tables, rigorous data‐quality checks, and a robust harmonization plan, remain highly relevant to CDM‐based studies. Adopting a CDM can streamline certain processes by enforcing standardized vocabularies and schemas, yet it can also introduce new challenges [17].

There is immense value in thorough communication and strong alignment across teams. This is exemplified by the feasibility checks and stepwise implementation outlined above. Minor discrepancies in understanding or implementation can snowball into significant issues later, as demonstrated by our previous studies with variable exclusion criteria, differences in event coding, or reimbursement policies that affect treatment access. When overseeing a multi‐national/database study, we strongly advocate that you always follow up immediately on any discrepancy or uncertainty that you encounter. It will save you time in the long run.

Multinational studies may also have additional administrative challenges, such as negotiating research agreements, handling data processing agreements under General Data Protection Regulation (GDPR), and managing data availability lags or retrieval delays that we may not have covered in depth here. While these legal and logistical concerns are important, we consider the ten focus areas presented above to be the most critical ones. It is also important to note that this paper is not intended to be a systematic review of all existing multinational/database studies or a survey of principal investigators; rather, our focus is on sharing the practical lessons learned from challenges and solutions we encountered through direct experience and illustrative examples. We have personally fallen into each of these pitfalls more than once. Nevertheless, a dedicated follow‐up study that systematically reviews every multinational/database real‐world observational database study, supplemented by targeted surveys of their lead investigators to capture the methodological challenges they encountered, would be invaluable for identifying best practices and common pitfalls across the field.

An African proverb says, “If you want to go fast, go alone. If you want to go far, go together”. Coordinating a multi‐national/database study in pharmacoepidemiology may seem complex and challenging, but with careful planning, thorough communication, and a structured approach, it's entirely achievable—even for young researchers. Each key step outlined here, from creating detailed metadata tables to ensuring consistent protocol implementation across sites and aligning result presentation and interpretation, could be used to build a strong foundation for your study, and researchers can navigate these challenges effectively.

Multi‐national/database studies offer unique opportunities to generate high‐quality, generalizable evidence, and with careful planning and proactive problem solving, their potential can be fully realized.

### 5.1 Plain Language Summary

Multi‐national/database studies on the use and effects of medicine are getting more popular for answering important health questions, such as understanding differences in treatment outcomes and medication safety across countries. However, conducting these studies can be challenging because healthcare systems, data collection methods, and coding practices vary from place to place. This paper provides 10 practical considerations to overcome these challenges and ensure reliable results. It highlights the importance of describing each dataset in detail, defining medical terms consistently, and maintaining clear communication between collaborators. Key steps include reviewing the usefulness of available data by for example, comparing treatment patterns across databases and using consistent methods for selecting and analyzing participants. Examples show how problems such as differences in how illnesses are recorded or how treatments are prescribed can be solved with careful planning. Conducting international studies on medicine use and safety can be challenging, but it becomes manageable with careful planning, clear teamwork, and solving problems as they arise.

## Conflicts of Interest

Kenneth Man reports grants from the CW Maplethorpe Fellowship, the European Union Horizon 2020, the UK National Institute of Health Research, the Hong Kong Research Grant Council, the Hong Kong Innovation and Technology Commission, and personal fees from IQVIA, unrelated to the current work. Anton Pottegård reports participation in research projects funded by Alcon, Almirall, Astellas, Astra‐Zeneca, Boehringer‐Ingelheim, Novo Nordisk, Servier, and LEO Pharma, all regulator‐mandated phase IV studies, all with funds paid to the institution where he was employed (no personal fees). Further, he has received one unrestricted grant from Novo Nordisk, unrelated to the current work.
